# The Uptake of Rare Trace Elements by Perennial Ryegrass (*Lolium perenne* L.)

**DOI:** 10.3390/toxics11110929

**Published:** 2023-11-15

**Authors:** Hayley Jensen, Niklas Lehto, Peter Almond, Sally Gaw, Brett Robinson

**Affiliations:** 1 School of Physical and Chemical Sciences, University of Canterbury, Christchurch 8041, New Zealandsally.gaw@canterbury.ac.nz (S.G.); 2 Department of Soil and Physical Sciences, Lincoln University, Lincoln 7647, New Zealand; niklas.lehto@lincoln.ac.nz (N.L.); peter.almond@lincoln.ac.nz (P.A.)

**Keywords:** bioaccumulation coefficient, perennial ryegrass, phytomanagement

## Abstract

Technological development has increased the use of chemical elements that have hitherto received scant scientific attention as environmental contaminants. Successful management of these rare trace elements (RTEs) requires elucidation of their mobility in the soil–plant system. We aimed to determine the capacity of *Lolium perenne* (a common pasture species) to tolerate and accumulate the RTEs Be, Ga, In, La, Ce, Nd, and Gd in a fluvial recent soil. Cadmium was used as a reference as a well-studied contaminant that is relatively mobile in the soil–plant system. Soil was spiked with 2.5–283 mg kg^−1^ of RTE or Cd salts, representing five, 10, 20, and 40 times their background concentrations in soil. For Be, Ce, In, and La, there was no growth reduction, even at the highest soil concentrations (76, 1132, 10.2, and 874 mg kg^−1^, respectively), which resulted in foliar concentrations of 7.1, 12, 0.11, and 50 mg kg^−1^, respectively. The maximum no-biomass reduction foliar concentrations for Cd, Gd, Nd, and Ga were 0.061, 0.1, 7.1, and 11 mg kg^−1^, respectively. Bioaccumulation coefficients ranged from 0.0030–0.95, and increased Ce < In < Nd ≅ Gd < La ≅ Be ≅ Ga < Cd. Beryllium and La were the RTEs most at risk of entering the food chain via *L. perenne*, as their toxicity thresholds were not reached in the ranges tested, and the bioaccumulation coefficient (plant/soil concentration quotient) trends indicated that uptake would continue to increase at higher soil concentrations. In contrast, In and Ce were the elements least likely to enter the food chain. Further research should repeat the experiments in different soil types or with different plant species to test the robustness of the findings.

## 1. Introduction

New technology uses Rare Trace Elements (RTEs), which have previously not been emitted into the environment at high concentrations. Soil contamination via the disposal of mining and extraction waste, rudimentary recycling of electronic waste [1], and leaching of materials from landfills has increased rapidly with the industrial use of RTEs [2,3,4,5]. Beryllium, Ga, In, La, Ce, Nd, and Gd are RTEs of concern that are used across several industrial sectors at the rate of tens or hundreds of tonnes per year [6].

Upon entry to soil, these RTEs either become associated with the solid phase through specific or non-specific adsorption to organic or inorganic matter, precipitation, or remaining in solution [7]. In the soil solution, RTEs may leach downwards through the soil profile or be taken up by plants [8]. Ions associated with the solid phase of the soil age to less soluble forms the longer they are in soil [9]. As RTEs are not known to be essential for plants and because soil background concentrations are relatively modest (Table 1) [10], plants are unlikely to have evolved specific mechanisms affecting tolerance or uptake. In soil, soluble RTEs will migrate toward plant roots via mass flow and diffusion [11]. Inside the root cortex, they are transported to the xylem tissues via the apoplastic or symplastic pathways [12]. Transportation across membranes into the symplast may occur, via the pathways of chemically similar ions [11], e.g., Ga^3+^ may be taken up via the same mechanism as Fe^3+^ in strategy II plants because the ions are similar in size and electronegativity [13]; La, Ce, Nd, and Gd can replace Ca^2+^ [14]; and Be can replace Ca^2+^ and Mg^2+^ [15]. Once in the roots, RTEs may be translocated around the plant, and if they enter the above-ground biomass or the edible organs, animal and human ingestion may occur. If RTEs are retained by soil, they can be ingested through soil retained on the surfaces of plant material [16], which is more common in erosion-prone areas where the contaminated soil can migrate, and on root crops such as potatoes that are directly in contact with the contaminated soil. RTEs may be taken up and translocated by plants if they are present in the soil solution, such as in hydroponic systems or acidic soils where elements such as Ga and In have not been hydrolysed to insoluble forms [17,18,19]. In hydroponic conditions, rice seedlings contained 74 mg kg^−1^ Ga without toxicity [18]. The phytotoxicity of these elements when added to soil depends on solid-phase retention and thus bioavailability, which vary between soil types [17,20,21]. Bioaccumulation coefficients (BACs), defined as the plant/soil concentration quotient for RTEs, typically range from <0.1–1.0 [17,22,23,24,25], but in rare instances can be >1.0 in plants which have strategies to take up and mitigate the phytotoxicity of RTEs, such as wild species growing in contaminated locations in Khan et al. [26].

There is a lacuna in studies comparing plant uptake and bioaccumulation of RTEs to each other and to common contaminants such as Cd. For example, the concentrations of RTEs in *Fagus sylvatica* leaves increased In < Gd < Be < Ga < La < Nd < Ce, from 0.0002 to 0.066 mg kg^−1^ [27], and in Browntop grass Gd < Be < Nd < La < Ce, from 0.0031–0.025 to 0.051–0.13 mg kg^−1^ [28]. Bioaccumulation coefficients in wheat/barley stems and grains increased Nd < La < Ga < Be < Ce, ranging from 0.0022–0.48 in stems and 0.00053–0.14 in grains [20]. However, the plants in the aforementioned studies were grown in uncontaminated soils. In contaminated soils, the RTEs may be less strongly sorbed to soil and thus more bioavailable [9]. Two studies used contaminants, e.g., root–shoot translocation of In was higher than Ga in Vietnamese wild plants growing in contaminated soil [3], and *L. perenne* growing in spiked soil had a higher uptake and bioaccumulation of Ga than In [29], but these studies only measured two of the selected RTEs, and did not compare the solubility to that of other contaminants.

We hypothesised that the uptake of the RTEs Be, Ga, In, La, Ce, Nd, and Gd by *L. perenne* growing in spiked soil would be proportional to the elements’ soluble concentrations in the soil, consistent with other elements for which there is no specific uptake mechanism [27,30]. We aimed to determine the uptake of *Lolium perenne* with Be, Ga, In, La, Ce, Nd, and Gd in a greenhouse trial, and compared their behaviour to a well-studied soil contaminant (Cd).

## 2. Materials and Methods

### 2.1. Soil Preparation

A fluvial recent soil, typical of a relatively high-fertility agricultural soil was collected from Christchurch (−43.5229981 S, 172.5873929 E). Surface litter was removed using a spade and soil collected from the top 15 cm. The soil was homogenised using a spade. The soil was an acidic (pH 5.3) loamy sand (81% sand, 16% silt, 3% clay), with high Olsen P (27 mg kg^−1^), a moderate cation exchange capacity (13 cmol kg^−1^), and low concentrations of organic C (1.9%). Table 1 shows the concentrations of RTEs (determined using the methods in 2.3) in the soil. The soil had stones removed and was dried and sieved to <2 mm. Note that there was significant variation in background concentrations due to the soil’s parent material [31] so our results differ somewhat to the global background reported by Kabata-Pendias and Mukherjee [10].

Soil was spiked with RTEs at rates proportional to their background concentrations (Table 1), specifically the treatments comprised 5 (T1), 10 (T2), 20 (T3), and 40 (T4) times the reference RTE and Cd-background concentrations, with the RTEs and Cd spiked in separate samples. The spiked concentrations were relative to the background concentrations of the RTEs and Cd in soil, as they varied by orders of magnitude, from 0.255 mg kg^−1^ In to 56.6 mg kg^−1^ Ce, thus the addition of the same concentrations of the elements would almost certainly cause toxicity by some elements and have no effect on others, and the aim was to identify the toxicity thresholds of each element (toxicity calculations explained later), and find how *L. perenne* takes up and translocates each element, relevant to what it has evolved to.

Cadmium and all RTEs were added as nitrate salts, except for Be, which was added as sulphate. While nitrogen and sulphur affect plant growth and metal uptake [32], the amounts added in these experiments were low compared to the amounts already present in a typical soil [33]. All RTEs except Cd and Be were in the +3 oxidation state. The mass of salt required to achieve the RTE or Cd concentration in T4, in 2 kg of soil, was solubilised in 100 mL of deionised water, which was transferred to a spray bottle and made up to approximately 200 mL. The solution was evenly incorporated into 2 kg of soil; 1 kg was taken out, and 250 g of that soil was placed into three pots, and 1 kg of uncontaminated soil was added to the remaining 1 kg of spiked soil to create a dilution. Dilutions were repeated to prepare each treatment. Three pots with uncontaminated soil were used as a control. The pots were square, with a height of 100 mm, a diameter of 70 mm across the top, and 50 mm across the bottom. Three replicates of each sample type were placed in a shared saucer to prevent contamination between sample types from water, and the big saucers were placed in a randomised design in a greenhouse. Pots were watered, and the RTEs and Cd were left to equilibrate for seven weeks.

### 2.2. Plant Growth

*Lolium perenne* was used for this experiment. *Lolium perenne* is grown in temperate areas of Asia, Australia, Europe, New Zealand, North America, South Africa, and South America. It is a member of the Poaceae family, and thus shares physiological similarities to wheat, barley, and the other grains which are present in this family, which are important food crops [34,35]. While some grasses, growing on metalliferous soils, have evolved tolerance to soil contamination [36], *L. perenne* used in agriculture has no reported adaptations for metal contamination. The leaves of *L. perenne* (representing >90% of the aboveground biomass) accumulate non-essential elements such as Cd at concentrations proportional to their respective soluble concentrations in soil [32,37]. We chose this species for the experiments because, due to its widespread use for pastoral production, RTE uptake could result in the entry of potentially toxic elements into the food chain. On the 9th of October, 2019, 50 seeds of *L. perenne*, variety Nui, obtained from Luisetti seeds, Rangiora, were sown into each pot. Pots were watered three times a week for 15 min each time for the duration they were in the greenhouse, from 8 overhead sprinklers at a rate of 0.47 L h^−1^ for each sprinkler. One week later, most of the seeds had germinated. The *L. perenne* was harvested on the 17th of December 2019. During the growth period, the day length varied from 14 h (October) to 16 h (December). Minimum temperatures ranged from 4 °C to 12 °C and maximum temperatures ranged from 18 °C to 29 °C. The aboveground portions of *L. perenne* were cut 1 cm above the soil, rinsed in distilled water, placed into separate paper bags, and dried at 65 °C until a constant weight was obtained. The dried material was stored in zip-lock bags until analysis.

### 2.3. Digestion and Measurement

In 15 mL glass vials, 0.05–0.5 g of dried *L. perenne* was added to 5 mL of concentrated HNO_3_ and left overnight. The next day, the samples were digested in a Milestone UltraWAVE single reaction chamber microwave digestion system at 220 °C for 25 min using the General digestion method for environmental samples. After they had been cooled and stored, the samples underwent a 21× dilution in 10 mL of ultrapure 1% HNO_3_. The samples were then analysed using inductively coupled plasma mass spectrometry, which measured the concentrations of Be, Na, Mg, Al, P, K, Ca, Cr, Mn, Fe, Co, Ni, Cu, Zn, Ga, As, Cd, In, Te, La, Ce, Nd, Gd, and Pb (listed in order of atomic number). The concentrations of the elements in *L. perenne* were all reported on a dry weight (DW) basis. The National Institute of Standards and Technology [38] Certified Reference Material was used, and the measurements were 60–140% of the certified value.

Soil digestions were completed in the same way, with 0.2–0.3 g of soil added to concentrated HNO_3_ and digested, to measure the pseudo-total concentration (Table 1), and Ca(NO_3_)_2_ extractable concentrations were determined using the method in [39].

### 2.4. Data Analysis

Bioaccumulation coefficients (BACs) were calculated by dividing the concentration of the RTEs or Cd in each of the *L. perenne* samples (mg kg^−1^ DW) by the concentration of the RTE or Cd in soil. In the treatments, the total concentrations of the elements in soil were assumed to be the pseudo-total concentration in the soil (Table 1) plus the concentrations added to the treatments which were gained from [10].

Biomass indexes were calculated by dividing the mass of the *L. perenne* in each sample against the average mass in the control. When the biomass indexes of the treatments were significantly lower than the control, it was assumed that adding the RTEs and Cd to soil reduced the biomass production of *L. perenne*, and thus phytotoxicity occurred. The toxicity threshold for each RTE was established as the concentration at which the plant biomass was significantly lower than the control (as determined by least significant differences).

The biomass indexes for the RTEs and Cd at the same concentration added to soil were calculated by adding trendlines to graphs with the concentration added to soil versus BAC, and inputting the same concentration added to soil into the trendlines for each of the elements.

Data in Supplementary Materials were tested for normality. Log-normally distributed data were log-transformed before analysis. A single-factor ANOVA analysis was performed and the least significant differences (LSD) were calculated. Elements were compared using a Pearson correlation analysis. The threshold for significance was *p* < 0.05.

## 3. Results

### 3.1. Effect of RTEs and Cd on the Biomass of L. perenne

There was a significant reduction in biomass in the treatments containing Cd (T1 and above), Ga (T3 and above), Ce (T1 and above), Nd (T3 and above), and Gd (T3 and above) (Table 2). However, for Be (T2) and Gd (T1) the biomass was significantly higher than in the control. There were no differences in biomass in the In and La treatments. The toxicity (as indicated by a reduction in biomass) of Cd and the RTEs decreased Cd > Gd > Nd > Ga, and the maximum concentrations of Be, Ce, In, and La did not cause consistent significant reductions to biomass within the range tested. The maximum concentrations of the RTEs in *L. perenne* found in this study without reductions to biomass (thus T4 or the highest treatment without reductions in growth) increased Cd < In < Gd < Be < Nd < Ga < Ce < La on a mass basis, and the ranking was similar on a molar basis. In the plants with reduced biomass, only Cd showed visible signs of chlorosis, and no plants had visible necrotic tissue.

### 3.2. Uptake and bioaccumulation of RTEs in L. perenne

In the control, the concentrations of the RTEs and Cd in *L. perenne* increased In < Cd < Be < Cd < Gd < Ga < Nd < La on a mass basis, and In < Cd < Ce < Gd < Nd < La < Ga < Be on a molar basis. Lanthanum, Ga, and Nd were taken up at the highest concentrations in the control and in all the treatments on a mass basis, and on a molar basis Ga, Be, and La were taken up at the highest concentrations. Indium consistently had the lowest uptake by *L. perenne*, with In concentrations of 0.05 mg kg^−1^ in the control, and a maximum of 0.11 mg kg^−1^ In in T4 when 10.2 mg kg^−1^ In was added to soil.

**Figure 1:**
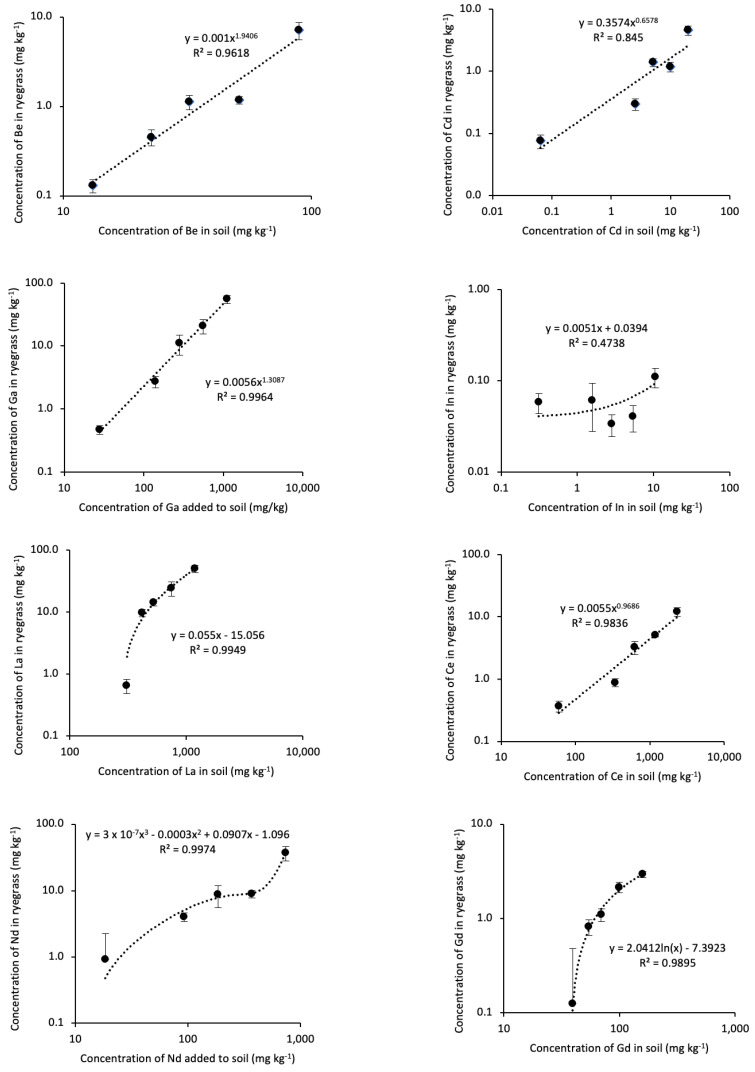
Concentrations of Be, Cd, Ga, In, La, Ce, Nd, and Gd in the shoots of ryegrass growing in contaminated soil.

Apart from In, uptake of the RTEs and Cd by *L. perenne* increased with increasing concentrations of the RTEs and Cd added to soil (Figure 1). The uptake of Be and Cd in T4 was high and did not fit the trendlines well. *Lolium perenne* Ga and La concentrations increased linearly with the concentrations of these elements added to soil. The uptake of Nd had a sigmoid response, which like Be and Gd is due to the inordinately high uptake of these elements in T4.

**Figure 2:**
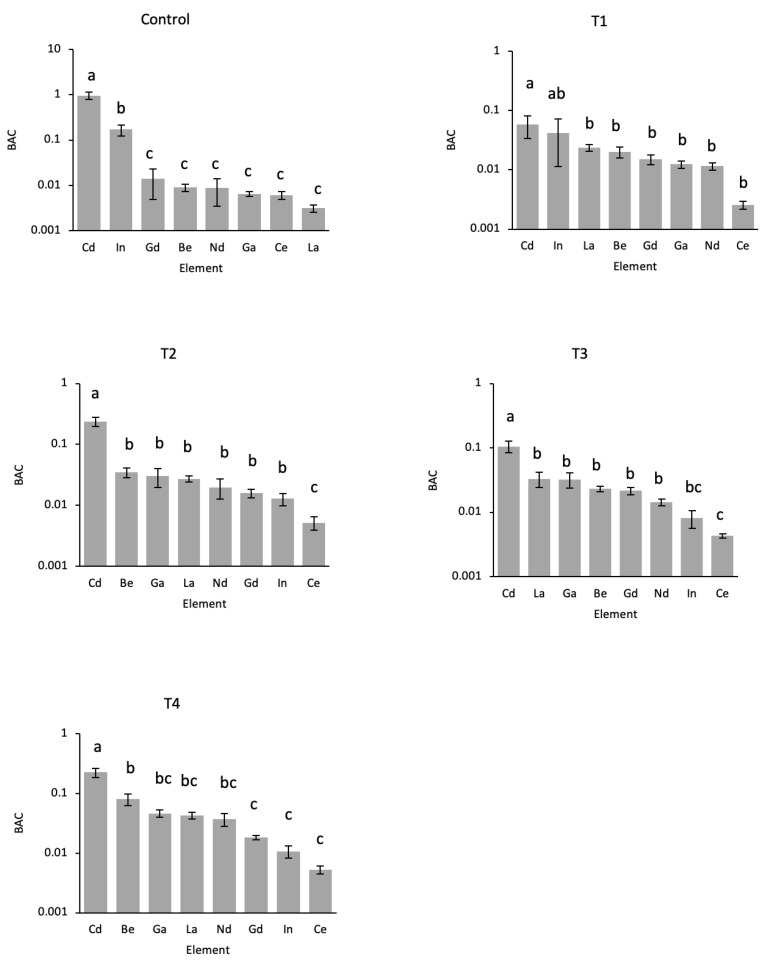
Bioaccumulation coefficients for the uptake of Be, Cd, Ga, In, La, Ce, Nd, and Gd in *L. perenne* grown in soil spiked with none (**Control**), 5 (**T1**), 10 (**T2**), 20 (**T3**), and 40 (**T4**) times the reference RTE and Cd-background concentrations. Bars labelled with the same letter are not significantly different.

BACs of the RTEs and Cd in *L. perenne* varied from 0.0031–0.95 in the control (Figure 2) to 0.0052–0.22 in T4 (Figure 2). Across all the treatments, BACs increased Ce < In < Nd≅Gd < La ≅ Be ≅ Ga < Cd. When BACs were calculated at the same concentration of RTE or Cd added to soil, at 5 mg kg^−1^ RTE or Cd added to soil, the ranking of the RTEs and Cd was the same as T1 except Gd had a higher BAC than Be and La, and at 1 mg kg^−1^ added to soil, the ranking of the BACs was La < Nd < Ce < Ga < Gd < In < Be < Cd.

The concentration of Be in *L. perenne* was significantly positively correlated with the concentrations of Al, Cd, Gd, La, Mg, Na, Nd, and Zn (Table 3). The concentrations of Gd, In, and La taken up by *L. perenne* did not affect the uptake of many of the other elements in *L. perenne*.

## 4. Discussion

### 4.1. Effect of the RTEs and Cd Contaminants on the Biomass of L. perenne

Cadmium, Ga, Nd, and Gd significantly reduced the biomass of *L. perenne*. In our experiment, the Cd concentrations in *L. perenne* were below the 3–30 mg kg^−1^ plant Cd toxicity threshold range reported by other authors (Table 4), but the concentration of Cd added to soil in T1 was close to the soil Cd toxicity threshold of 5 mg kg^−1^ [40]. The soil used in this study was an acidic sandy loam (pH 5.3) with a moderate cation exchange capacity (13 cmol kg^−1^), and thus was expected to have high solubility and bioavailability of RTEs and Cd [17,21], which may have contributed to the low soil toxicity threshold of Cd. For the other elements, whether they experienced toxicity or not in this study, the toxicity thresholds were close to the ranges in Table 4 for Be, Ga, In, La, Ce, and Nd. The concentrations of the RTEs added to soil which induced toxicity were similar to other studies [41,42,43,44,45]. The addition of Be and Gd to soil significantly increased the biomass of *L. perenne*, likely due to hormesis, an increase in growth in response to stress [46], as both elements are non-essential to plants, and the biomass consistently decreased from the peak when higher concentrations of these elements were present in the soil. The anions added to the soil as the counterion in the RTE and Cd salts added to soil, SO_4_^2−^ and NO_3_^−^, may have also increased growth in the treatments.

For Be, Ce, La, and In, no toxicity occurred within the range tested. The soil and conditions (e.g., pH) in this study may have caused high retention of some elements, but not others, e.g., Ga and In precipitate and thus are bioavailable at different pH ranges in soil [47]. Future research should test the relative phytotoxicity of Be, Ce, La, and In at higher soil concentrations and in different soil types.

**Table 4:** Plant concentrations of the RTEs in unspiked and spiked soils.

Element	Plant Concentrations (mg kg^−1^) in Unspiked Soil		Uptake in Plants Spiked with ETECs (mg kg^−1^, μmol kg^−1^)	
	Background	Elevated (without Visible Toxicity)	Before Toxicity	With Toxicity
Be	Lettuce: 0.06 [23] Grass: 0.0051 [28] Wheat grain: <0.0005 [48]	Potato foliage: 30 [41] Collards foliage: 10 [44] Oat foliage: 2 [41]	0.1–10.1, 11–1121 [44,49]	0.4–32, 44–3551 [49,50]
Ga	Strawberry: 0.4 [51] Beech leaf: 0.032 [27] Wheat grain: 0.0014 [48]	Rice foliage: 75 [18] Wheat foliage: 12.1 [21] Mushroom: 6.6 [52]	2–74, 29–1061 [18,53]	5–16, 72–229 [17,21]
In	Wheat grain: 0.11 [54] Grass: 0.051 [24] Beech leaf: 0.0002 [27]	Mushroom: 7.5 [55] Pteris vittata: 5.14 [3] Rice foliage: 4.3 [17]	1.1–4.2, 9.6–37 [17,18]	2.5–6.6, 22–57 [17,21]
La	Grass: 0.17 [56] Tea: 0.072 [57] Various vegetables: 0.017 [58]	*Cyperus rotundus* L.: 568.9 [26] Barley foliage: 6 [59] *Populus sieboldii*: 3.4 [57]	0.44–6.5, 3.2–47 [43,60]	0.95–120, 6.8–864 [43,45]
Ce	Grass: 0.33 [56] Pepper: 0.19 [22] Corn: 0.011 [61]	*Dicranopteris dichotoma*: 2290.33 [26] Rice foliage: 19 [25] Chinese cabbage: 3.8 [25]	6.7, 48 [60]	16, 114 [60]
Nd	Grass: 0.051 [62] Rice: 0.029 [63] Tomato: 0.0071 [64]	*Melastoma malabathricum*: 49.13 [26] Mushroom: 7.1 [65] Barley foliage: 7 [59]	1.79–13, 12–90 [42]	6.69–221, 46–1532 [42,45]
Gd	Grass: 0.025 [28] Pepper: 0.015 [22] Barley grain: 0.0003 [48]	*Cyperus rotundus* L.: 175.8 [26] *Dicranopteris dichotoma*: 75.08 [26] Rice foliage: 0.49 [25]	-	-

The ‘background’ column consists of examples of the concentrations of RTEs typically found in various plant species in environments without contamination, and the ‘elevated’ column has examples of unusually high uptake of the RTEs in plants without toxicity.

### 4.2. Uptake of the RTEs and Cd in L. perenne

The ranking of the uptake of the RTEs and Cd by *L. perenne* differed from the ranking of RTE accumulation by *Fagus sylvatica*, which may be due to the physiological differences between a monocotyledonous plant (*Lolium perenne*) and a dicotyledonous plant (*Fagus sylvatica*) [28]. Cerium was hypothesised to be taken up at the highest concentrations by *L. perenne*, but was only the fourth highest, with the lowest BACs of the RTEs and Cd. Compared to the ranking of the RTEs in [27], the uptakes of Ga and La were higher than the other RTEs. The uptake of the RTEs in *L. perenne* was similar to that (within the same order of magnitude) of grasses and other members of the Poaceae family [10,56,61]; it is useful to compare within this family if possible, as they have physiological differences to other plant species which affect uptake and translocation, e.g., they are monocotyledonous plants, and use Strategy II for Fe acquisition [66], which helps them to acquire higher concentrations of Cu, Mn, and Zn [67]. In studies that spiked soil with similar concentrations of the RTEs, the uptakes of Be and Ga in *L. perenne* were similar to that in oat plants, collards, cabbage, wheat, and rice seedlings [17,21,41,44,68].

The grasses (*L. perenne* and *Leersia hexandra*) growing in soil that had been contaminated previously (with sufficient time for residential development and wild species to grow) contained concentrations of Ga and In, which were more similar to the control versus the grass growing in contaminated soil [24]. This suggests that ageing quickly reduced the bioavailability of the contaminants, or that the most bioavailable fraction was quickly taken up by plants and passed up the food chain. Thus, the concentrations of the RTEs found in *L. perenne* in this study are only comparable for environments where recent contamination has occurred. Our study therefore represents a ‘worst-case’ scenario.

### 4.3. Bioaccumulation Coefficients of the RTEs and Cd in L. perenne

The BAC values were, for the most part, similar to those of Poaceae species [56,59,69], in contaminated and uncontaminated soil. BACs for In in *L. perenne* were higher than that for In in rice and wheat [54], but higher concentrations of In were added to the soil in that study, which is consistent with the BAC of In decreasing with increasing concentrations added to soil.

### 4.4. Effect of the Concentration of RTEs and Cd Added to Soil on Bioaccumulation in L. perenne

The literature indicates that Cd has a relatively high BAC because it is more soluble in soil than other trace elements [70], which has been verified by the lesser solid-phase retention [20]. Plant uptake of Cd linearly increased between the control and T4. Therefore, it seems unlikely that the uptake and translocation of Cd was limited by the plants.

Cadmium, a ‘soft’ Lewis acid, binds more strongly to soft functional groups such as S^2-^ than hydroxide groups [71]. The soft functional groups may have been present at high concentrations in soil, which Cd bound to, limiting the availability of exogenous Cd to plants, particularly at the lower concentrations of Cd added to soil. This was not directly measured in the soil properties however, so it cannot be confirmed.

The change in the BACs with increasing soil concentrations was different for Ce and Gd than La and Nd. In the control, Ce and Gd had higher BACs than La and Nd and thus had higher uptakes than expected relative to the sum of the REEs in the soil. It cannot be explained why the bioavailability of endogenous Ce and Gd was higher than endogenous La and Nd, and the existing high uptake may have limited the increase in bioaccumulation factors.

Another process that may have increased the uptake of the elements is following the uptake pathways of essential ions, which would result in a significant negative correlation with the essential ion, as the RTE would occupy spaces in the molecules responsible for the uptake and translocation of essential elements. There were few negative correlations across the elements (Table 3), and those often occurred with anions (Ga and La with P, Cd with As and I) which the cations would not substitute for in biological molecules. The uptake of Be was positively correlated with the uptake of Mg, but this could be a strategy to ameliorate toxicity by taking up higher concentrations of essential elements to maintain the physiological processes which Be may disrupt [15]. Beryllium was one of the RTEs that did not cause toxicity within the range tested in this study, which supports the above theory. Two of the four REEs had significant positive correlations with Ca, to which the REEs have physiological similarities [14], but they were Ce and Nd, elements whose BACs were little affected and increased with the concentration added to soil, respectively. Thus, the significance of this correlation on plant uptake is unknown. The uptake of Be was positively correlated with uptake of the REEs, thus Be stress may increase the uptake of Mg and Ca, resulting in a net increase in growth.

The concentrations of each element added to the soil in the treatments were relative to the concentrations of the elements naturally in soil and may not represent concentrations in contaminated soils. If the same concentrations of each RTE and Cd were added to soil, at >750 mg kg^−1^ the BAC of Nd would be higher than Ga, Gd, and La, and Be would be higher than Cd if the patterns in Figure 1 extrapolate without saturation.

### 4.5. Risk of RTEs Entering the Food Chain

By discussing mobility, the relative risks of the RTEs entering the food chain can be evaluated. Of the RTEs and Cd tested in this study, Be and La have the highest risk of entering the food chain via *L. perenne*. Beryllium and La did not cause significant phytotoxicity within the concentration range tested, and the BACs of these elements increased with the concentration added to soil. Thus, the uptake of Be and La will likely continue to increase.

Only Cd, Ga, Nd, and Gd had toxicity thresholds, which occurred at plant concentrations of 0.3, 21, 9.0, and 2.1 mg kg^−1^, respectively. Thus, relative and non-relative to the background concentrations of the elements in soil, Be and La have the highest risk of being taken up and translocated to above-ground biomass at high concentrations by *L. perenne*.

Contrastingly, Cd had a higher BAC than the RTEs, but had a low toxicity threshold, with reductions to growth occurring in T1. The low toxicity threshold contrasts with previous findings of Cd being a mobile and easily transferable contaminant to animals [72]. Thus, the cultivar of *L. perenne* used (Nui) should be regrown in different environments to confirm its low tolerance to Cd. The RTEs least likely to be taken up and translocated by *L. perenne* (variety Nui) are In, as uptake did not increase with the concentration added to soil, and Ce, due to the low BACs. Cerium was safely taken up at the second-highest concentration in *L. perenne* due to the high concentrations naturally in and thus added to soil.

Unlike other crop types such as grains and vegetables, *L. perenne* does not need to survive reproduction or be harvested to enter the food chain. In the field, reductions in *L. perenne* growth by RTEs could be mistaken for symptoms of drought, nutrient deficiency, disease, or insufficient aeration, increasing the feasibility of animals eating contaminated *L. perenne*. Currently, there are no food safety standards in place for these RTEs, and thus the implications of animals ingesting the *L. perenne* grown in this study are unknown.

## 5. Conclusions

The addition of Be, Ce, In, and La to soil at 40x the background concentration did not cause significant reductions to the biomass of *L. perenne*. Cadmium and Gd had the lowest toxicity thresholds of the selected RTEs. The bioaccumulation coefficients of the RTEs and Cd increased Ce < In < Nd≅Gd < La ≅ Be ≅ Ga < Cd. The elements with the highest overall mobility in the soil–plant system are La and Be, as they were present at 50 and 7.1 mg kg^−1^, respectively, in *L. perenne* in the highest treatment, and the BACs of these elements are likely to continue to increase beyond the range tested. The least mobile elements in the variety of *L. perenne* tested were Cd, as it had a low toxicity threshold; Ce, as it had low BACs; and In, as its uptake did not increase with the concentration added to the system. Therefore, data in Supplementary Materials showed that there is a low risk of *L. perenne* uptake being a significant source of Cd, Ce, and In to animals, but there is a higher risk of *L. perenne* being a source of Be and La to animals and humans. Further research should determine whether the trends identified in this study are repeatable across a range of soil types, plant species, and climates, to better evaluate the mobility of these RTEs in the soil–plant system. Then the rates of transfer of these elements to animals and their ecotoxicology should be determined.

## Figures and Tables

**Table 1:** Pseudo-total (conc. HNO_3_-extractable) concentrations of the Rare Trace Elements and Cd used for reference in the treatments. Values in brackets represent the standard error of the mean (n = 5).

Element	Concentration in Experimental Soil (mg kg^−1^)
Be	13 (0.55)
Cd	0.064 (0.0018)
Ga	89 (1.1)
In	0.31 (0.0081)
La	308 (1.5)
Ce	60 (0.53)
Nd	256 (4.0)
Gd	39 (0.56)

**Table 2:** Treatment RTE and Cd concentrations, biomass indexes, and plant toxicity thresholds gained from biomass index significance for *L. perenne*. Brackets show standard error to the mean. Treatments with the same letter are not significantly different, from a (lowest biomass index) to c (highest).

RTE or Cd Added to Soil	Treatment and Concentration Added (mg kg^−1^)		Biomass Index	Significance	Toxicity Threshold in Plant Biomass	
					(mg kg^−1^)	(μmol kg^−1^)
Be	Control	0	1	ab	>7.1	>790
	T1	9.5	1.1 (0.28)	abc		
	T2	19	1.9 (0.52)	c		
	T3	38	1.6 (0.053)	bc		
	T4	76	0.56 (0.11)	a		
Cd	Control	0	1	c	0.06–0.30	0.54–2.6
	T1	2.5	0.70 (0.046)	b		
	T2	5	0.81 (0.078)	bc		
	T3	10	0.76 (0.012)	b		
	T4	20	0.47 (0.11)	a		
Ga	Control	0	1	bc	11–21	159–300
	T1	140	1.3 (0.27)	c		
	T2	280	0.83 (0.061)	ab		
	T3	560	0.56 (0.056)	a		
	T4	1120	0.69 (0.060)	ab		
In	Control	0	1	a	>0.11	>0.98
	T1	1.275	1.1 (0.42)	a		
	T2	2.55	1.0 (0.20)	a		
	T3	5.1	1.3 (0.20)	a		
	T4	10.2	0.84 (0.064)	a		
La	Control	0	1	ab	>50	>362
	T1	109.25	0.88 (0.18)	ab		
	T2	218.5	1.5 (0.25)	b		
	T3	437	1.2 (0.43)	ab		
	T4	874	0.71 (0.22)	a		
Ce	Control	0	1	b	>12	>87
	T1	283	0.37 (0.058)	a		
	T2	566	0.87 (0.081)	b		
	T3	1132	0.85 (0.22)	b		
	T4	2264	0.84 (0.22)	b		
Nd	Control	0	1	c	8.7–9.0	60–62
	T1	92.25	0.80 (0.019)	bc		
	T2	184.5	0.75 (0.10)	abc		
	T3	369	0.51 (0.041)	a		
	T4	738	0.72 (0.15)	ab		
Gd	Control	0	1	b	1.1–2.1	6.0–14
	T1	15.075	1.7 (0.31)	c		
	T2	30.15	0.58 (0.045)	ab		
	T3	60.3	0.44 (0.069)	a		
	T4	120.6	0.69 (0.025)	ab		

**Table 3:** Correlation *r* values of significant correlations among the concentrations of Be, Cd, Ga, In, La, Ce, Nd, or Gd in *L. perenne* grown in soil spiked with the respective element and the controls, and the concentrations of other elements in *L. perenne*. S = significant (0.05 > *p* > 0.01), S* = highly significant (0.01 > *p* > 0.001), S** = very highly significant (*p* < 0.001).

	Be	Cd	Ga	In	La	Ce	Nd	Gd
Be								
Na	0.50 S		0.48 S				0.48 S	
Mg	0.69 S**					0.52 S*		
Al	0.67 S**							
P			−0.54 S*		−0.47 S			
K								
Ca						0.53 S*	0.51 S	
Cr						0.54 S*		
Mn		−0.53 S*						
Fe		−0.14 S				0.62 S*	0.60 S*	
Co			0.58 S*			0.56 S*	0.47 S	
Ni		0.59 S*				0.58 S*		0.53 S*
Cu						0.49 S	0.42 S	
Zn	0.78 S**	0.44 S	0.58 S*			0.55 S*		
Ga				0.50 S				
As		−0.82 S**						
Cd	0.49 S		0.57 S*		0.50 S*	0.50 S	0.42 S	0.45 S
In								
Te								
I		−0.76 S**						
La	0.49 S		0.52 S			0.65 S**	0.72 S**	
Ce							0.64 S**	
Nd	0.44 S		0.58 S*	0.37 S		0.77 S**		
Gd	0.41 S		0.53 S*			0.79 S**	0.56 S*	

## Data Availability

Elemental concentrations in plant materials are available on: https://www.kiwiscience.com/journal-articles.html (accessed on 12 November 2023).

## Supplementary Materials

The following supporting information can be downloaded at: https://www.mdpi.com/article/10.3390/toxics11110929/s1.
